# The Owen Bill up Again; Emasculated

**Published:** 1912-05

**Authors:** 


THE OWEN BILL UP AGAIN; EMASCULATED.—The Senate Committee on Public Health has reported favorably on this bill as amended to meet the absurd objections on the part of the League for Medical Freedom. The bill is on the calendar as No. 561. The ground has been cut from under the feet of the "freedom shriekers" by inserting in the bill a distinct provision that it shall *not* "interfere in any way with any of the functions belonging to any State," that *no* one shall be authorized to "enter the premises of private persons without permission of the owner or occupant"; that it shall *not* "interfere with or seek to regulate the practice of medicine or the practice of healing"; it shall *not* interfere with the "right of a citizen to employ the doctor of his choice"; it shall *not* make any "discrimination in favor of any school of medicine or healing," etc.

What will the fanatics do now? They will trump up some objection, and deluge Congress with telegrams.

The bill provides for a United States Public Health Service to supersede the M. H. S.,—under a Director of Health, to be appointed by the President, and absorbs or consolidates, under this Department, all the several "Bureaus" in the several Departments, towit: Vital Statistics from the Department of Commerce and Labor; that of Chemistry from the Department of Agriculture, which is charged with the enforcement of the Pure Food and Drugs Law. There will be seven bureaus and divisions, under the new Department of Health, namely: a Bureau of Public Health and Marine Hospital Service; a Bureau of Foods and Drugs; A Bureau of Vital Statistics; a Bureau of Child Conservation, and divisions of Sanitary Engineering; Personnel and Accounts and Publications, etc.

It is to be hoped now that the Committee of One Hundred and the A. M. A. will resume activities, and use every legitimate means to secure the passage of even this mutilated act; for the Senate will not act favorably unless it can be shown that public sentiment demands it. Friends of the bill used, all in vain, the marvelous results of sanitation in Cuba and in the Canal Zone, in an effort to get a better bill passed—one that creates a Department of Public Health with a regular physician and sanitarian at the head, such as Gorgas, for instance.

Well, it is better than no bill, and is perhaps an improvement on the existing arrangement, in which all health matters are scattered through several departments as "bureaus." But it is eminently unsatisfactory, and far short of what the medical profession desire and what is needed. The bars have been let down and all kinds of cattle can enter; and, when some demagogue is President, we may expect to see a Christian Science "healer" appointed "Director of the Public Health," or an Osteopath, Homeopath—or some other "path" at the head of each subdivision.

What's the use? "Against stupidity [and venality], even the gods are powerless."

P. S.—Even now, already, the Christian Science Senator, Work, of California, is reasserting in a speech that the bill will do all the things which it specifically states that it will *not* do! What's the use?

